# Comment on Sung et al. Body Fat Reduction Effect of *Bifidobacterium breve* B-3: A Randomized, Double-Blind, Placebo Comparative Clinical Trial. *Nutrients* 2023, *15*, 28

**DOI:** 10.3390/nu15051093

**Published:** 2023-02-22

**Authors:** Sang Yeoup Lee

**Affiliations:** 1 Family Medicine Clinic, Biomedical Research Institute, Pusan National University Yangsan Hospital, Yangsan 50612, Republic of Korea; saylee@pnu.edu; Tel.: +82-55-3601442; 2 Integrated Research Institute for Natural Ingredients and Functional Foods, Yangsan 50612, Republic of Korea; 3 Department of Medical Education, Pusan National University School of Medicine, Yangsan 50612, Republic of Korea

I read with interest the paper by Sung et al. entitled “Body Fat Reduction Effect of *Bifidobacterium breve* B-3: A Randomized, Double-Blind, Placebo Comparative Clinical Trial” where a reduction in body fat mass after *Bifidobacterium breve* B-3 (BB-3) ingestion for 12 weeks was reported [1]. Despite the presentation of alternative treatment results that have advanced the treatment of obesity in this paper, additional information is required to be applied effectively and appropriately to the reader’s clinical practice.

In this study, 50 billion colony-forming units (CFUs) were consumed per day, but people generally consume various lactic acid bacteria through different foods. For Asians, various fermented foods, such as kimchi, chongkukjang, doenjang, ganjang, and gochujang, are sources of lactic acid bacteria. Additionally, various yogurts contain plenty of lactic acid bacteria. Therefore, it seems that those who had taken lactic acid bacteria within the past month were excluded, but it was not mentioned whether or not participants took lactic acid bacteria during the study period. Furthermore, each person’s basic intestinal flora may be different, and changes in the intestinal flora may occur after BB-3 administration [2]. If the authors intestinal flora data, it would be useful to know these clinical results. Second, the dual-energy X-ray absorptiometry (DEXA) used by the author was a fan-beam type and is known to be able to analyze body fat relatively accurately. However, along with other blood tests, the DEXA should present the results of the equipment’s accuracy and precision. Bilsborough et al. [3] reported that its precision value for fat-free soft tissue mass was excellent at 0.3% (coefficient of variation, %CV), and the acceptable reliability of fat mass and percent body fat were both 2.5% (%CV). At 12 weeks, the difference in body fat mass between the BB-3 group and the placebo group of 35.99 g (Table S4) appears to be within the 2.5% reported in Bilsborough et al. [3], suggesting no significant difference. Moreover, at week 12, the BB-3 group lost an average of 1.08 kg compared to the placebo group (Table S2), and only body fat mass was significantly different in body composition analysis by DEXA. Furthermore, body fat mass decreased significantly more in the BB-3 group than in the placebo group, but there is no clear explanation for the lack of difference in body fat percentage between the two groups after 12 weeks. To better aid readers’ understanding, it would be helpful if the authors presented the %CV values of precision and acceptable reliability for this study, as these values may differ between studies. Third, a previous study by Minami et al. in 2015 [4] revealed that body fat was reduced by 1.1 kg via a daily administration of 50 billion CFUs, the same amount administered in this paper. However, in a study conducted in 2018 by Minami et al. [2], 20 billion CFUs per day, less than half of this amount, was administered, yet still resulted in a significant body fat reduction. Presenting dosage determination data would be beneficial.

There was also something interesting concerning safety. The study mentioned the side effects of post-COVID-19 vaccination in study subjects. Recently, it has been reported that there is a change in body weight after the COVID vaccine [5] Thus, information on the COVID-19 vaccine administration during the study period between the two groups is necessary. Additionally, an intention-to-treat (ITT) analysis result considering 12 dropouts would be more useful than the per-protocol (PP) analysis result in clinical practice. Results 3.4. Physical Activity and Diet revealed that there was no difference in diet and exercise changes between the two groups for 12 weeks (Table S8), yet no analysis of the differences between diet and exercise at the beginning of the study was found. Furthermore, Minami et al. [2] explained the mechanism of BB-3’s body fat reduction as the up-regulation of Cyp7A1 expression in the liver, which is different from the authors’ suggestion. Lastly, suggesting the limitations of the study would be helpful for follow-up research.
